# Identifying chronic obstructive pulmonary disease from integrative omics and clustering in lung tissue

**DOI:** 10.1186/s12890-023-02389-5

**Published:** 2023-04-11

**Authors:** Brian D Hobbs, Jarrett D Morrow, Xu-Wen Wang, Yang-Yu Liu, Dawn L DeMeo, Craig P Hersh, Bartolome R Celli, Raphael Bueno, Gerard J Criner, Edwin K Silverman, Michael H Cho

**Affiliations:** 1 Channing Division of Network Medicine, Brigham and Women’s Hospital, Harvard Medical School, 181 Longwood Ave, Rm 460, Boston, MA 02115 USA; 2 Division of Pulmonary and Critical Care Medicine, Brigham and Women’s Hospital, Harvard Medical School, Boston, MA USA; 3 Division of Thoracic Surgery, Brigham and Women’s Hospital, Boston, MA USA; 4 Division of Pulmonary and Critical Care Medicine, Temple University School of Medicine, Philadelphia, PA USA

**Keywords:** Chronic obstructive pulmonary disease, Gene expression, DNA methylation, Integrative omics, Clustering, Similarity network fusion, Entropy-based consensus clustering

## Abstract

**Background:**

Chronic obstructive pulmonary disease (COPD) is a highly morbid and heterogenous disease. While COPD is defined by spirometry, many COPD characteristics are seen in cigarette smokers with normal spirometry. The extent to which COPD and COPD heterogeneity is captured in omics of lung tissue is not known.

**Methods:**

We clustered gene expression and methylation data in 78 lung tissue samples from former smokers with normal lung function or severe COPD. We applied two integrative omics clustering methods: (1) Similarity Network Fusion (SNF) and (2) Entropy-Based Consensus Clustering (ECC).

**Results:**

SNF clusters were not significantly different by the percentage of COPD cases (48.8% vs. 68.6%, p = 0.13), though were different according to median forced expiratory volume in one second (FEV_1_) % predicted (82 vs. 31, p = 0.017). In contrast, the ECC clusters showed stronger evidence of separation by COPD case status (48.2% vs. 81.8%, p = 0.013) and similar stratification by median FEV_1_% predicted (82 vs. 30.5, p = 0.0059). ECC clusters using both gene expression and methylation were identical to the ECC clustering solution generated using methylation data alone. Both methods selected clusters with differentially expressed transcripts enriched for interleukin signaling and immunoregulatory interactions between lymphoid and non-lymphoid cells.

**Conclusions:**

Unsupervised clustering analysis from integrated gene expression and methylation data in lung tissue resulted in clusters with modest concordance with COPD, though were enriched in pathways potentially contributing to COPD-related pathology and heterogeneity.

**Supplementary Information:**

The online version contains supplementary material available at 10.1186/s12890-023-02389-5.

## Background

Chronic obstructive pulmonary disease (COPD) is a common and progressively worsening disease characterized by airflow limitation and unrelenting respiratory symptoms including shortness of breath, cough, and sputum production [1]. Additionally, COPD is one of the leading causes of death worldwide, accounting for 3.17 million deaths in 2015 [2]. One of the primary difficulties in treating persons with COPD is the clinical heterogeneity of the disease [3–5]. Although COPD is diagnosed using spirometry, two patients with similar lung function impairment may have vastly different clinical features [6]. Some COPD therapies are only effective (or indicated) in patients with specific clinical features [7–9]. The development of new therapies and improved prognostication in COPD may depend on the identification of specific subtypes or sub-phenotypes in COPD [10]. Clinical clustering is one way to identify previously unrealized subtypes of COPD, and these clinically similar subtypes may be enriched for specific biologic processes, as evidenced by differential COPD genetic risk loci enrichment within clinical COPD subtypes [11]. Clustering using omics data may lead to the description of endotypes (biologically-based subtypes) of COPD patients sharing common dysregulated pathways. Clustering of single omics, particularly with biologic and clinical constraints, such as the clustering of blood gene expression data selected based on lung function and constrained on the protein-protein interaction network via network-based stratification, is one alternative to clustering on clinical features alone [12].

Multiple omics integration and clustering may offer the opportunity to consider the broader pathobiologic processes that explain the COPD clinical heterogeneity. Several investigators have already explored this possibility. Li and colleagues applied the multi-omic integration method Similarity Network Fusion (SNF) [13] across up to 9 overlapping omics data types in a cohort of 52 female never-smokers, current smokers with normal spirometry, and current smokers with mild-to-moderate COPD [14]. In their study, Li et al. described improving subgroup classification accuracy with accuracy > 95% when using different combinations of 4 to 7 omics data types, even with decreasing sample size. Whether these results can be seen in lung tissue, with the integration of a fewer number of omics data types, is not clear. A study of a multi-omics subtyping in 489 participants in the COPDGene study utilized autoencoders to generate reduced-dimension embeddings of blood-based transcriptomics, proteomics, and metabolomics data [15]. The reduced-dimension representations of the omics data were then either integrated “early” and subsequently clustered or were integrated “late” after clusters were generated from the single-omics autoencoder embeddings. The authors reported advantages and disadvantages to both early and late integration of omics data for subtyping without finding a superior approach for all hypotheses [15]. It remains unclear to what extent COPD is reflected in gene expression and methylation data of lung tissue and whether known clinical subtypes (such as spirometry-defined airflow limitation) are the best ground truth for unsupervised clustering of omics data for the purpose of discovering novel COPD subtypes.

We evaluated two integrative omics and clustering methods, SNF (as previously used by Li, et al.) and Entropy-based Consensus Clustering (ECC), to determine whether COPD case status and COPD heterogeneity are captured in lung tissue omics data. SNF generates summary measurements of patient-to-patient similarity for each omics data type and then integrates the patient similarity networks into a final fused network, upon which clustering can be applied [13]. By contrast, the ECC method is based on generating hundreds of potential clustering solutions directly from integrated omics data with a final clustering solution generated by consensus across the hundreds of potential clustering solutions [16]. We applied the SNF and ECC methods to genome-wide gene expression and methylation data in a lung tissue cohort of former-smoking severe COPD cases and controls. From our resulting unsupervised clustering we sought to (a) determine the concordance of the clusters with spirometry-defined COPD status and other COPD-related phenotypes and (b) identify molecular characteristics of the clusters.

## Methods

### Lung tissue cohort

Lung tissue samples were collected at the time of either lung transplant, lung volume reduction surgery, or lung mass removal (including wedge resection, segmentectomy, lobectomy, or pneumonectomy) at one of three clinical centers including Brigham and Women’s Hospital (Boston, MA), St. Elizabeth’s Medical Center (Brighton, MA), and Temple University Hospital (Philadelphia, PA). The specific surgical indication and type of surgery performed to collect each lung tissue sample was not recorded. In the case of lung nodule or mass resection, adjacent normal tissue was selected for analysis. Subjects provided written informed consent and Institutional Review Board approval was obtained at all three clinical centers. All subjects were former smokers with at least one month of abstinence from smoking prior to thoracic surgery. Available phenotype data included demographics, anthropometrics, cigarette smoking history, spirometry, and pulmonary emphysema and airway thickening measurements from quantitative computed tomography (CT) imaging. Given the nature of the indications for thoracic surgery and the sample ascertainment protocol, subjects had either normal or severely impaired lung function. Lung tissue samples were labeled as controls if they had normal lung function defined by forced expiratory volume in one second (FEV_1_) ≥ 80% of predicted and a ratio of FEV_1_ to forced vital capacity (FVC) ≥ 0.7. Conversely samples were labeled as severe COPD cases if they had FEV_1_ < 50% of predicted and FEV_1_/FVC ratio < 0.7. Additional cohort details have been previously described [17, 18].

### Gene expression and DNA methylation profiling

Lung tissue samples were snap frozen and stored at -80 °C prior to RNA and DNA extraction from homogenized lung tissue using the AllPrep kit (Qiagen, Valencia, CA). RNA quality was assessed on a BioAnalyzer (Agilent, San Diego, CA). Gene expression profiling was performed on the lung tissue samples using the HumanHT-12 BeadChip (Illumina, San Diego, CA) assay as previously reported [17]. DNA methylation profiling for the lung tissue samples was performed on genome-wide CpG using the Infinium HumanMethylation450 BeadChip array (Illumina, San Diego, CA) as previously reported [18]. The gene expression and methylation profiling was obtained on the same lung tissue sample for each individual in the study. Information on the gene expression and methylation data quality control procedures can be found in the Supplementary Materials.

### Similarity network fusion (SNF) method summary

As the optimal method to cluster non-cancer samples is not clear, we chose to apply two complementary methods. Both methods required a pre-specified expected number of clusters. To determine how well multiple omic integration and clustering were able to differentiate lung tissue from former smoking controls (normal spirometry) and severe COPD (GOLD stage 3–4 spirometry), we set the expected number of clusters (k) to 2. First, we employed SNF [13] and spectral clustering [19] through the SNFtool (v 2.3.0) package in R to find lung tissue subtypes from integrated gene expression and methylation profiling. In brief, the SNF algorithm gives a final fused sample-to-sample similarity matrix representing the shared information from gene expression and methylation data. Additional details of the SNF approach can be found in the Supplementary Materials. Spectral clustering was then applied to the similarity matrix to identify k = 2 clusters [19]. We used normalized mutual information (NMI) [20, 21] to assess the similarity of clustering solutions from gene expression alone, methylation alone, and combined expression and methylation data. NMI equal to 1 denotes identical clustering solutions.

### Entropy based consensus clustering (ECC) method summary

For comparison to SNF, we employed an alternate multiple-omic integration and clustering method, ECC which was implemented in Matlab using the code published with the manuscript [16]. As with SNF, we set the expected number of clusters from ECC to be k = 2. In brief, the ECC method iteratively generates clustering solutions (basic partitions) with varying numbers of clusters and then relies on an entropy-based utility function to create consensus across the basic partitions to give a final clustering solution. The details of the application of ECC to our data can be found in the Supplementary Materials. Our final ECC clustering solution resulted in k = 2 clusters from consensus across 30,000 basic partitions (15,000 each for the gene expression and methylation data). As with SNF, we used the NMI to assess the similarity of clustering solutions from gene expression alone, methylation alone, and combined expression and methylation data.

### Evaluation of clinical differences between SNF and ECC clusters

For the clustering solutions provided by SNF and ECC, we compared the clinical characteristics of the individuals from which the lung tissue samples were obtained. Clinical features included age, sex, race, body mass index (BMI), pack years of cigarette smoking, and months since cessation of smoking cigarettes. We also evaluated spirometric values of the samples including percent of predicted forced expiratory volume in 1 s (FEV_1_pp), the ratio of FEV_1_ to forced vital capacity (FEV_1_/FVC), and COPD defined by FEV_1_/FVC < 0.7 and FEV_1_pp < 80%. Quantitative computed tomography (CT) characteristics available for comparison included percent emphysema measured by the % of low-attenuation areas (LAA) less than − 950 Hounsfield units (%LAA < -950 HU), severity of emphysema measured by the HU value at the 15th percentile of the lung density histogram (Perc15), and wall area thickness as measured by the square root of the wall area of a theoretical airway with a 10 mm internal perimeter (Pi10). Categorical variables were reported as N and percent and were compared with the Chi-squared test. Quantitative variables were compared using t-tests if normally distributed or Wilcoxon rank-sum tests if non-normal. The statistics (both descriptive and comparative) were generated in the tableone (v 0.10.0) package in R. For differences between any two clinical features, a P value of < 0.05 was considered statistically significant.

### Differential gene expression and pathway-based analysis

We performed a differential expression analysis to compare the gene expression signatures of the final SNF and ECC clusters, adjusting for age, sex, race, and pack-years of cigarette smoking. Differential expression analysis was performed in the limma (v 3.40.2) [22] package in R Bioconductor. Multiple testing penalty was applied using the Benjamini-Hochberg method with a false discovery rate (FDR) < 5% considered significant. For pathway enrichment, we considered a broader set of transcripts, evaluating differentially expressed transcripts between SNF clusters and between ECC clusters at an FDR < 10%. We assessed for enrichment of transcripts in Reactome Knowledgebase [23] pathways using the ReactomePA (v 1.32.0) [24] package in R Bioconductor. Reactome pathways were considered enriched with FDR < 5% in over-representation analysis.

### Sensitivity analysis for clustering on COPD case status

We assessed if selecting COPD-associated gene expression and methylation probes prior to implementing ECC would allow improved stratification of COPD cases in ECC clusters. We defined COPD classification accuracy as the number of COPD cases in the cluster with the larger percentage of COPD cases plus the number of controls in the other cluster divided by 78 (the total number of lung tissue samples subjected to clustering). For instance, if one cluster has 35 lung tissue samples, of which 24 are COPD and the second cluster has 43 samples of which 22 are controls, the COPD classification accuracy would be (24 + 22)/78 = 0.59 or 59%. We used previously published COPD differential expression [17] and differential methylation [18] results from the same lung tissue cohort and created subsets of transcript probes and methylation probes at COPD differential expression and differential methylation false discovery rate (FDR) thresholds of 5%, 10%, 25%, and 50%. We compared the ECC solutions using FDR-filtered transcripts and probes with the ECC solution obtained using genome-wide expression transcripts and methylation probes.

## Results

### Lung tissue cohort characteristics

We studied resected lung tissue from 78 former-smoking individuals with overlapping single-batch DNA methylation data and single-batch gene expression array data. Forty-five (58%) of the lung tissue samples were from severe COPD cases with 24% median (IQR 19–34%) FEV_1_% predicted and 32% quantitative emphysema on CT chest. There were no differences between controls and COPD cases in age, sex, and race. BMI was slightly lower in the COPD cases and time since quitting smoking was shorter in the COPD cases. As expected, COPD cases had lower lung function (FEV_1_% predicted and FEV_1_/FVC ratio), greater cigarette smoke exposure, and more quantitative emphysema (Table 1).

**Table 1:** Demographic, clinical, and imaging features of the 78 lung tissue samples with overlapping DNA methylation array and gene expression array data

Feature	Control	COPD Case	p
N (%)	33 (42%)	45 (58%)	
Age, years	65 (9.5)	64.1 (7.6)	0.63
Male Sex, N (%)	11 (33.3)	22 (48.9)	0.25
Caucasian Race, N (%)	29 (87.9)	44 (97.8)	0.20
BMI, kg/m^2^	28.53 (6.13)	25.97 (4.80)	0.043
FEV_1_% Predicted	97 [87, 106]	24 [19, 34]	< 0.001
FEV_1_/FVC Ratio	0.79 [0.74, 0.82]	0.28 [0.24, 0.39]	< 0.001
Pack-Years Smoking	33.30 (20.79)	58.16 (25.22)	< 0.001
Months Since Quit Smoking	143 [31, 291]	72 [24, 112]	0.020
%LAA < -950 HU (% emph)	0.01 [0.00, 0.07]	0.32 [0.18, 0.39]	< 0.001
HU at 15th % Lung Density Histogram (perc 15)	-909.17 (35.9)	-966.10 (30.1)	< 0.001
Pi10	3.98 (0.44)	4.21 (0.39)	0.16

Unless otherwise indicated values are either mean (standard deviation) or median [interquartile range].

*P value is from t-test for variables expressed as mean (sd) and Wilcoxon rank sum test for variables expressed as median [IQR].

BMI = body mass index; LAA = lung attenuation area; HU = Hounsfield units; Pi10 = square root of cross-sectional area of hypothetical 10 mm internal perimeter airway.

### Similarity network fusion (SNF) with spectral clustering application

We applied SNF our pre-processed and normalized lung tissue expression and methylation data, choosing the “optimal” set of hyperparameters (number of neighbors = 30, scaling parameter for sample similarity [a] = 0.8, SNF iterations = 15) to maximize variance across both the expression and methylation patient-to-patient similarity matrices. We repeated the SNF and spectral clustering procedure 10 times and the resulting cluster sizes and membership were the same with each trial. The resulting lung tissue clusters from SNF and spectral clustering are shown in Table 2.

**Table 2:** Demographic, clinical, and imaging features of two clusters identified from SNF applied to the 78 lung tissue samples with overlapping DNA methylation array and gene expression array data

Feature	SNF Cluster 1	SNF Cluster 2	P value
N (%)	43 (55%)	35 (45%)	
Age, years	66.1 (9)	62.4 (7.3)	0.055
Male Sex, N (%)	18 (41.9%)	15 (42.9%)	1
Caucasian Race, N (%)	42 (97.7%)	31 (88.6%)	0.24
BMI, kg/m^2^	26.8 (4.6)	27.3 (6.5)	0.73
FEV_1_% Predicted	82 [33, 100]	31 [20.5, 82.5]	**0.017**
FEV_1_/FVC Ratio	0.72 [0.29, 0.78]	0.33 [0.25, 0.71]	0.087
COPD, N (%)	21 (48.8%)	24 (68.6%)	0.13
Pack-Years Smoking	49.5 (29.8)	45.4 (21.6)	0.50
Months Since Quit Smoking	120 [35, 228]	60 [17.5, 108]	**0.044**
%LAA < -950 HU (% emph)	0.19 [0.02, 0.39]	0.20 [0.04, 0.33]	0.98
HU at 15th % Lung Density Histogram (perc 15)	-948.7 (43.3)	-947.6 (40)	0.92
Pi10	4.11 (0.38)	4.17 (0.46)	0.67

Unless otherwise indicated values are either mean (standard deviation) or median [interquartile range].

*P value is from t-test for variables expressed as mean (sd) and Wilcoxon rank sum test for variables expressed as median [IQR].

BMI = body mass index; LAA = lung attenuation area; HU = Hounsfield units; Pi10 = square root of cross-sectional area of hypothetical 10 mm internal perimeter airway.

### Evaluating SNF cluster stratification by COPD case status

Though median FEV_1_% predicted was significantly different between the two SNF clusters, COPD case status was poorly differentiated (48.8% vs. 68.6%, p = 0.13) with a COPD classification accuracy of 59% (Table 2). We also performed spectral clustering on the methylation and gene expression data alone and noted that the stratification of COPD case status from single ‘omics data (Supplemental Tables S1 and S2) was not superior to the clustering solution using the fused data from SNF. However, clustering on the expression data alone yielded two clusters significantly different in amount of quantitative emphysema measured by both %LAA < -950 HU and the HU value at the 15th percentile of the lung density histogram (Supplemental Table S1). Clustering on the methylation data alone yielded two clusters significantly different in median FEV_1_% predicted, though the between-cluster FEV_1_ difference (Supplemental Table S2) was less than observed for SNF applied to the integrated expression and methylation data (Table 2).

### Entropy-based consensus clustering (ECC) application

We then applied ECC to our lung tissue expression and methylation data. With 15,000 basic partitions and K = 2 (number of clusters) we reached a stable clustering solution. The resulting ECC clusters are shown in Table 3. The two ECC clusters were significantly different not only in FEV_1_% predicted and FEV_1_/FVC ratio, but also in COPD case status (48.2% vs. 81.8%, p = 0.013). In the smaller of the two ECC clusters, 18 (81.8%) of the 22 lung tissue samples are from persons with COPD. Despite the significant differences in spirometry values and COPD case status, there were no differences in amount of emphysema or airway wall thickness for the two ECC clusters. We compared the ECC results from the merged expression and methylation data to ECC solutions using the expression data and methylation data alone. ECC applied to gene expression data resulted in two clusters of lung tissue samples differing in the mean age and number of pack-years of smoking (Supplemental Table S3). The ECC solution for DNA methylation alone was identical (NMI = 1) to ECC applied to the merged data (Supplemental Table S4).

**Table 3:** Demographic, clinical, and imaging features of two clusters identified from ECC applied to the 78 lung tissue samples with overlapping DNA methylation array and gene expression array data (with 15,000 basic partitions for each data type)

Feature	ECC Cluster 1	ECC Cluster 2	P value
N (%)	56 (72%)	22 (28%)	
Age, years	64.9 (8.6)	63.3 (8.1)	0.43
Male Sex, N (%)	22 (39.3%)	11 (50%)	0.54
Caucasian Race, N (%)	54 (96.4%)	19 (86.4%)	0.26
BMI, kg/m^2^	26.7 (4.9)	27.8 (6.8)	0.43
FEV_1_% Predicted	82 [23.75, 99.5]	30.5 [20.5, 38]	**0.0059**
FEV_1_/FVC Ratio	0.71 [0.29, 0.78]	0.32 [0.24, 0.39]	**0.013**
COPD, N (%)	27 (48.2%)	18 (81.8%)	**0.014**
Pack-Years Smoking	46.1 (27.7)	51.5 (23)	0.42
Months Since Quit Smoking	112 [36, 201]	54 [14.3, 107]	0.057
%LAA < -950 HU (% emph)	0.17 [0.02, 0.38]	0.28 [0.11, 0.34]	0.50
HU at 15th % Lung Density Histogram (perc 15)	-946.1 (42.1)	-953.2 (40.6)	0.57
Pi10	4.10 (0.40)	4.20 (0.44)	0.55

Unless otherwise indicated values are either mean (standard deviation) or median [interquartile range].

*P value is from t-test for variables expressed as mean (sd) and Wilcoxon rank sum test for variables expressed as median [IQR].

BMI = body mass index; LAA = lung attenuation area; HU = Hounsfield units; Pi10 = square root of cross-sectional area of hypothetical 10 mm internal perimeter airway.

### Evaluating ECC cluster stratification by COPD case status

**Fig. 1:**
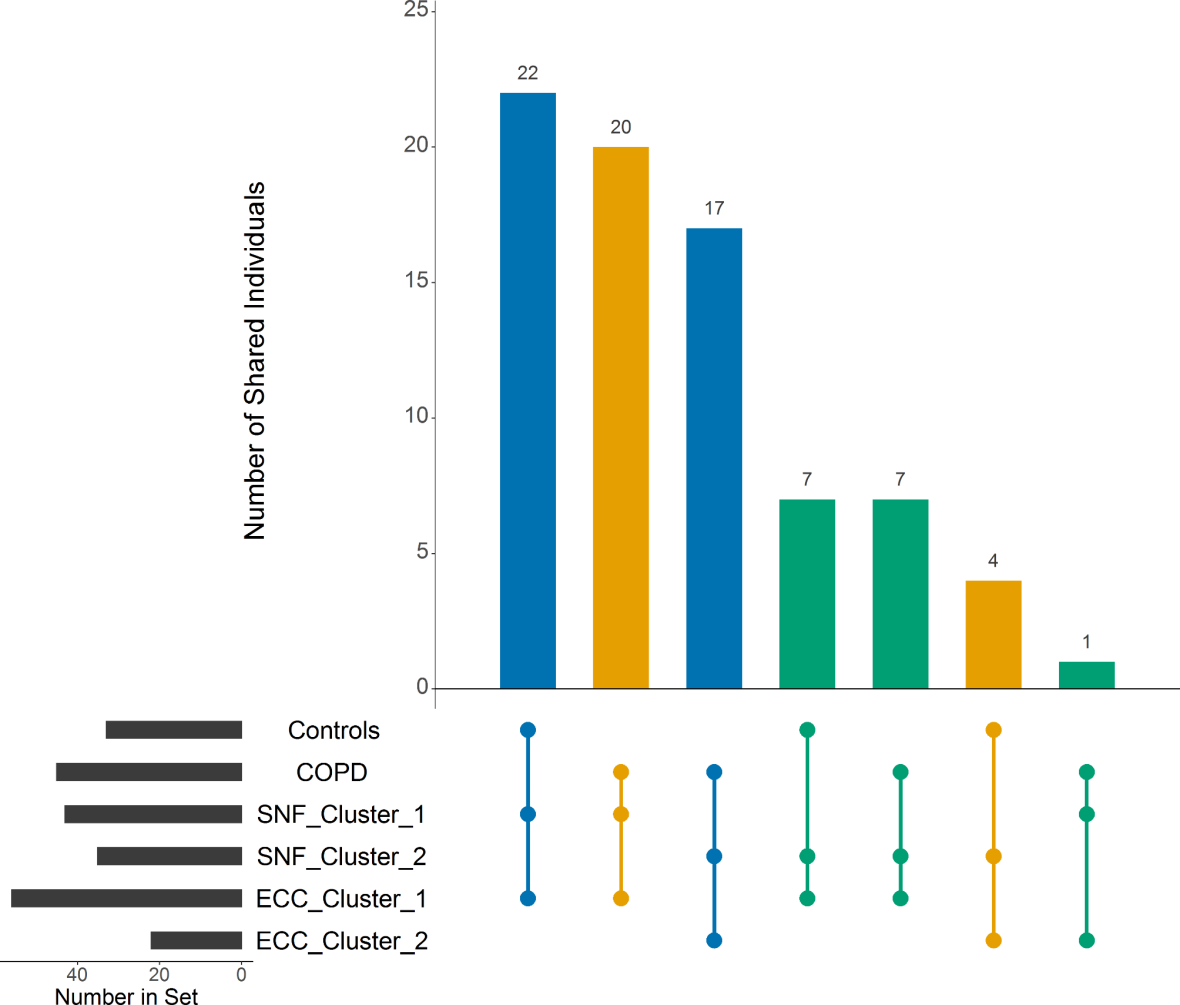
Overlap of SNF and ECC clusters with COPD case status. The Upset Plot illustrates the distribution of controls and COPD cases across the SNF and ECC clusters. Most individuals in SNF Cluster 1 and ECC Cluster 1 are controls while most individuals in SNF Custer 2 and ECC Cluster 2 are COPD cases. Blue bars indicate concordant clustering by SNF and ECC into “correct” clusters to align with COPD cases status. Orange bars indicate concordant clustering by SNF and ECC into “incorrect” clusters. Green bars indicate a disagreement between SNF and ECC clustering

The significant difference in the proportion of COPD cases between the two ECC clusters did not translate to substantive improvement in the overall COPD classification accuracy, which was 60% (29 controls in ECC Cluster 1 and 18 cases in SNF Cluster 2 correctly identified) compared to 59% for SNF. The distribution of controls and COPD cases across the SNF and ECC clusters can be seen in the Upset Plot (Fig. 1). ECC Cluster 2 contained only 4 Controls where SNF Cluster 2 contained 11 controls, driving a significant difference in COPD case status in the ECC clusters that was not observed in the SNF clusters. Therefore, as ECC was superior in identifying one cluster with a higher percentage of COPD cases, we performed additional analyses to evaluate the sensitivity of ECC’s clustering solutions to the input gene expression and methylation data.

**Fig. 2:**
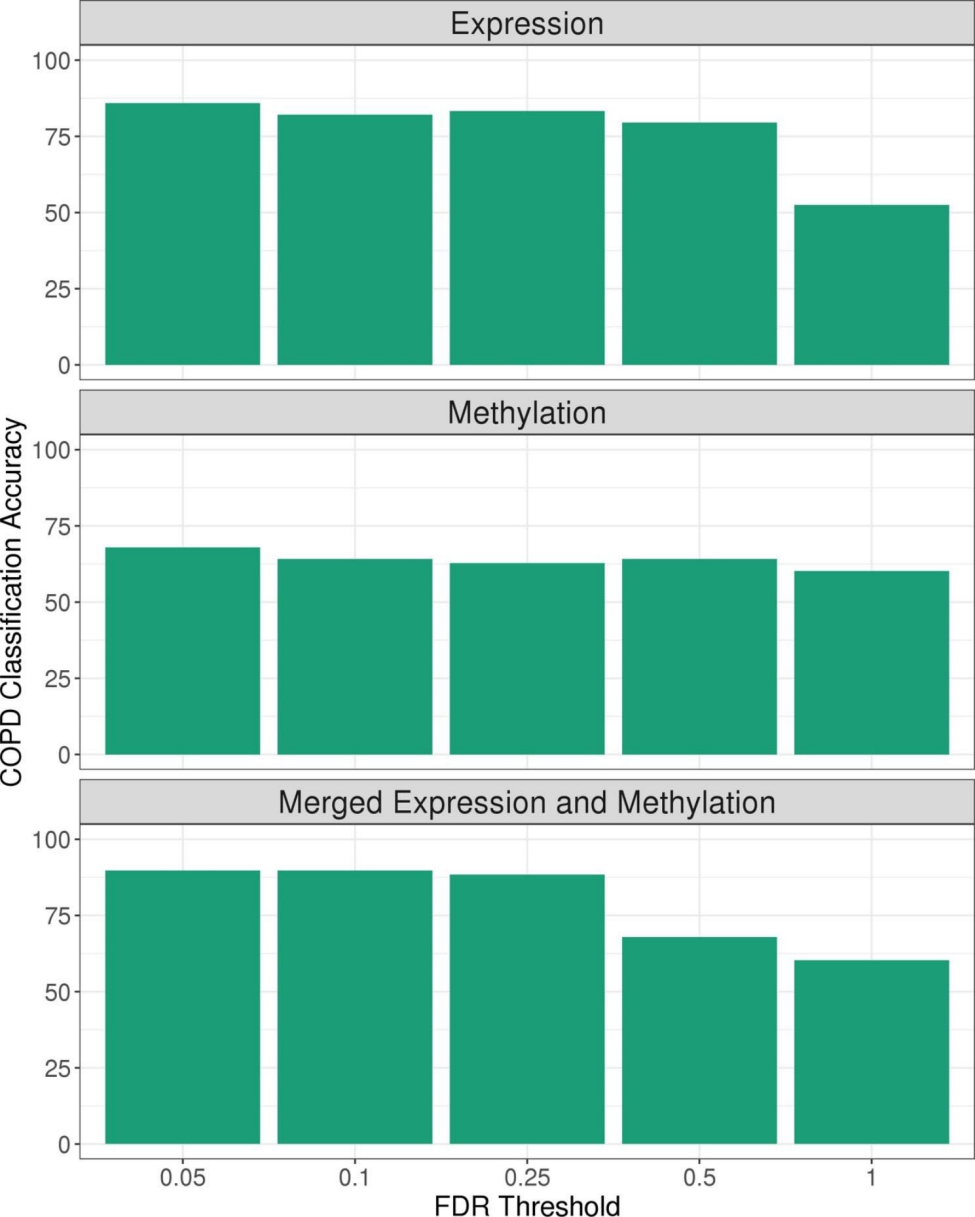
COPD Classification Accuracy for the two clusters generated from ECC applied to expression data alone, methylation data alone, and the integrated expression and methylation data. The expression and methylation input data were filtered according to false discovery rate (FDR) thresholds of COPD differential expression and COPD differential methylation, respectively. An FDR threshold of 1 indicates genome-wide data were used

To test whether ECC would be able to recover case control status using sets of differentially expressed (for COPD) transcripts and methylation probes previously identified in the same dataset, we ran ECC serially filtered to varying FDR cutoffs. The use of COPD differentially expressed transcripts at FDR < 0.05 resulted in an ECC classification accuracy of 86% compared to a classification accuracy of 53% using genome-wide expression data. With ECC applied to methylation data alone, the COPD classification accuracy was 68% when using probes with COPD differential methylation FDR < 0.05 compared to a 60% accuracy when using genome-wide methylation data. For the merged expression and methylation data, the COPD classification accuracy was 90% when using differentially expressed and differentially methylated data at FDR < 0.05 compared to an accuracy of 60% using merged genome-wide expression and methylation data (Fig. 2). When filtering ECC input data based on COPD differential expression or differential methylation at an FDR of 0.05, 0.1, and 0.25, the classification accuracy of merged data was slightly higher than the classification accuracy when clustering either expression data or methylation data alone. In contrast, with data filtered to an FDR of 0.5 the COPD classification accuracy of the expression data is superior to either methylation data alone or the merged data. Finally, if genome-wide expression and methylation data are used for ECC, the COPD classification accuracy of expression data alone nadirs to 53% and the clustering solution of methylation data alone is identical to the solution for merged data, with a COPD classification accuracy of 60%.

### Differential gene expression and pathway enrichment of SNF and ECC clusters

**Fig. 3:**
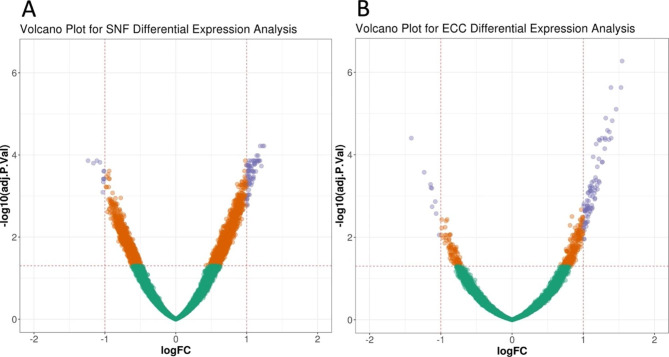
Volcano plots showing differentially expressed transcripts between lung tissue samples in the (A) final two SNF clusters and (B) final two ECC clusters generated from integrated lung tissue gene expression and methylation data. -log10(adj.P.Val) = the negative base 10 logarithm of the False Discovery Rate. logFC = log fold-change in gene expression values between clusters

We evaluated the differential expression of transcripts comparing lung tissue samples in the two final clusters generated by merged expression and methylation data in SNF and ECC. For the SNF clusters, 2856 transcripts were differentially expressed (FDR < 5%) with 80 transcripts having an absolute log-fold difference > 1 (Fig. 3A). The ECC clusters had 424 differentially expressed transcripts (FDR < 5%) including 96 transcripts with absolute log-fold difference > 1 (Fig. 3B). By way of comparison, differential expression analysis of COPD cases status in the same 78 lung tissue samples resulted in 9 differentially expressed transcripts, 8 of which had an absolute log-fold difference > 1.

Differentially expressed (FDR < 10%) SNF transcripts were enriched in 45 unique Reactome pathways with the immune pathways including “Neutrophil degranulation”, “Signaling by the B cell receptor”, “Toll-like receptor cascades”, “Signaling by interleukins”, and “Immunoregulatory interactions between a lymphoid and non-lymphoid cell” being some of the top pathways (Supplemental Table S5). Differentially expressed (FDR < 10%) ECC transcripts were enriched in five Reactome pathways including “Immunoregulatory interactions between a lymphoid and a non-lymphoid cell”, “Cell surface interactions at the vascular wall”, “Neutrophil degranulation”, “Signaling by Interleukins”, and “Generation of second messenger molecules” (Supplemental Table S6). With the exception of “Generation of second messenger molecules,” the enriched pathways from ECC differential expression were also enriched from the SNF differential expression data.

## Discussion

Gene expression and methylation have been extensively studied in COPD lung tissue, with reports of differentially expressed genes and methylation marks [17, 18, 25–30]. To determine whether these omics would be able to identify subtypes of COPD, we assessed two integration and clustering methods, SNF with spectral clustering and ECC, for the ability to recover molecular differences in lung tissue of severe COPD cases and controls with normal spirometry. In the application of these methods to COPD, a non-cancer polygenic complex disease, we had to make modifications to the recommended SNF and ECC parameters. For instance, to stably identify clusters using ECC, we expanded from the default 100 basic partitions (a.k.a. intermediate clustering solutions) to 15,000 partitions, a much more computationally intensive undertaking.

We expected two clusters in the integrated omics data to reflect spirometry-defined COPD case status, which is a marker for the greatest known phenotypic difference in the lung tissue samples. For example, COPD cases had median 32% emphysema and COPD controls had median 1% emphysema from quantitative CT measurements. Despite expectations, using integrated genome-wide expression and methylation data, both SNF and ECC clusters poorly recapitulated COPD case status; however, one of the ECC clusters was 82% COPD cases compared to the “best” SNF COPD cluster which contained 69% COPD cases. Our results indicate that COPD case status as defined by spirometry is not a major driver of gene expression and methylation differences in lung tissue.

SNF has been previously employed on a classification task in COPD. Li et al. used SNF to distinguish current-smoking COPD cases, current-smoking controls, and healthy never smokers and demonstrated that classification accuracy of SNF clusters is a function of the number of samples, the number of omics available for integration, and the specific types of omics data [14]. For any two of nine studied omics data types, Li et al. reported a mean accuracy of ~ 40% and a maximum accuracy of ~ 75%. In our study we demonstrated a 60% COPD classification accuracy with ECC (and 59% for SNF), which is within the range of expectation established by Li et al. and the lessons from SNF may apply more broadly to multiple omic integration and clustering. That said, several key differences between our study and that of Li et al. should be noted. We clustered using bulk gene expression and methylation profiling from lung tissue homogenate in contrast to the bronchoalveolar lavage (BAL) and plasma samples utilized in Li et al. The lung tissue samples in our study were obtained from former smokers with normal lung function and with severe-to-very-severe COPD (FEV_1_% predicted < 50%); the samples in Li et al. were from healthy never smokers compared, current smoking individuals with normal lung function, or current smoking individuals with mild-to-moderate COPD (FEV_1_% predicted > 50%). The difference is the smoking status between our two studies should be emphasized as smoking status has a large effect on both peripheral blood and lung tissue transcriptomic profiles [31–34].

We can also view the relatively poor overlap of our SNF and ECC clusters with COPD diagnosis in the context of data generated by the integrative phenotype framework (iPF) method developed by Kim et al. in 2015 and applied to 319 lung tissue samples from individuals with either COPD or interstitial lung disease (ILD) [35]. Kim et al. used iPF to generate a clustering solution from integration microRNA and RNA data as well as from high-dimensional clinical phenotype data utilizing 669 clinical variables, including lung function variables diagnostic for COPD. Although one cluster of 76 individuals was enriched for COPD cases and another cluster of 80 individuals was enriched for idiopathic pulmonary fibrosis (a specific sub-phenotype of ILD), and additional 7 clusters from 163 lung tissue samples had a mixture of COPD and ILD cases. The experience of Kim et al. further demonstrates the difficulty of recapitulating clinical diagnostic classifications using unsupervised clustering of multiple omic (and high dimensional phenotype) data.

SNF and ECC are optimized to define clusters based on the greatest molecular differences between samples, which may be determined by factors other than COPD case status. To assess the impact of gene selection on integrative omic clustering, our sensitivity analysis of ECC classification accuracy for COPD first filtered the input gene expression and methylation data based on the association of both data types with COPD and demonstrated that COPD classification accuracy was maximal (90%) when integrating the highest confidence (FDR < 5%) gene expression and methylation data. Classification accuracy slowly degraded as additional data were included up to only 60% COPD classification accuracy using genome-wide gene expression and methylation data. Gene expression and methylation data are inherently noisy, and our experience further illustrates the difficulty with identifying a replicable COPD disease signal from genome-wide omics data.

Though neither SNF and ECC recapitulated COPD case control status, both SNF and ECC performed well in defining clusters on their greatest molecular differences. There were marked gene expression differences (2856 and 424 differentially expressed genes for SNF and ECC, respectively) between the two clusters, particularly when compared to only nine differentially expressed genes for COPD case status in the same set of lung tissue samples. The gene expression differences between our clusters allow us to glean biologic insights about our clustering solutions. Despite the differences in the integration and clustering methods, there were consistent gene expression signals differentiating the two clusters generated by SNF and ECC. The gene expression signal differentiating our SNF and ECC clusters was enriched for immunoregulatory and inflammatory pathways. COPD is an inflammatory condition [36–38] and previous network-based studies of lung and blood transcriptomics have similarly implicated immune and immune-cell specific processes [39, 40]. Thus, our gene expression and methylation integration and clustering results are in line with prior COPD integrative omics and clustering data as well as previous biology of gene expression in COPD samples, suggesting pathways that potentially relate to disease activity.

Our study had several limitations. Although the lung tissue cohort we employed for this analysis has DNA methylation and gene expression data from ~ 160 individuals, our sample size was limited to 78 samples from single methylation and expression batches due to confounding of batches by COPD case status. Sample size directly contributes to statistical power as well as the success of machine learning tasks such as clustering and classification. It is unclear how much our clustering solution accuracy for COPD classification would have improved if we were able to use all ~ 150 lung tissue samples. Although there is precedent for identifying inflammatory signatures associated with COPD and COPD-related phenotypes in lung and blood gene expression studies, the association of our clusters with inflammation may be driven by non-COPD processes such as unmeasured operative variables (e.g. distance of sample from diseased tissue, length of surgery, time from resection to freezing, etc.) at the time of sample collection. Both our study and previous omics studies of lung tissue have likely failed to adequately control for factors related to lung tissue sample acquisition. We did not have access to detailed information on the indication for the surgery from which the lung tissue samples were obtained and there may be differences in omics signatures of lung tissues related to surgical indication or type. For instance, if a COPD case lung tissue sample was obtained from lung volume reduction surgery due to severe emphysema and a case was obtained from lobectomy in the setting of lung cancer, the variability in the two lung tissue samples may be driven more by surgical indication and type of surgery than by differences in spirometry. We also lack information on disease activity and longitudinal phenotype data, which may have allowed us to identify clusters of individuals at highest risk for disease progression. Another limitation involves the use of bulk lung tissue gene expression and methylation profiling, which is summative of the gene expression and DNA methylation profiles of greater than 40 different resident lung cells [41] as well as hematologic cells with an array of biologic functions. Several emerging methods such as Bisque [42] attempt to estimate cell type proportions in bulk tissue omics profiling; however, the success of these methods depends on high quality single cell RNA and methylation data to improve prediction of cell type composition of samples. Future work will explore adjustment for cell type heterogeneity prior to omics integration and clustering. Finally, the unsupervised clustering results presented in this manuscript should be externally validated in another lung tissue cohort to determine if: (1) the findings generalize to larger and more diverse populations of patients (as our study was restricted to three eastern United States clinical centers and comprised mostly European-ancestry individuals), and (2) if our biologic pathway enrichment findings highlighting the processes separating our lung tissue clusters can be confirmed. Subsequent work could then focus on developing functional studies to determine the specific drivers (including cellular heterogeneity) to explain the observed molecular differences separating lung tissue clusters in individuals with and without COPD.

Despite these limitations, our study again illustrates that spirometry-defined COPD is clinically heterogenous [4–6]. Gene expression and DNA methylation reflect active molecular processes in the lung. A substantial portion of COPD cases may have reduced maximal lung function early in life with normal lung function decline over time [43, 44]. Gene expression in these participants with reduced lung growth and normal decline may be more like gene expression in participants with normal spirometry than like participants with active disease contributing to rapid lung function decline. Our findings of clusters enriched for inflammatory signatures further emphasizes the role of differential activation of these pathways in former smokers, some of whom develop COPD.

## Conclusions

Using two integrative omic and clustering methods leveraged on genome-wide gene expression and methylation data in lung tissue from former smokers with normal lung function or with severe COPD generated clusters only moderately recapitulating COPD case status as defined by spirometry. However, the genes differentially expressed between clusters were enriched in pathways potentially contributing to COPD-related pathology and heterogeneity. Our study underscores some of the challenges in omics in COPD and calls for additional efforts in longitudinal data collection to differentiate disease activity from disease severity and novel phenotypes and biomarkers to further understand disease heterogeneity.

## Electronic supplementary material

Below is the link to the electronic supplementary material.

Supplementary Material 1

## Data Availability

The microarray dataset and corresponding phenotype data for this study can be found in the Gene Expression Omnibus (GEO accession GSE76925). The raw methylation data are not fully availability given privacy concerns regarding the release of the IDAT files; however, the beta matrix used in this analysis will be available from the authors upon reasonable request.

## Abbreviations

COPD: Chronic Obstructive Pulmonary Disease
SNF: Similarity Network Fusion
ECC: Entropy-based Consensus Clustering
CT: Computed Tomography
FEV_1_: Forced Expiratory Volume in one second
FVC: Forced Vital Capacity
RNA: Ribonucleic Acid
DNA: Deoxyribonucleic Acid
GOLD: Global Initiative for Chronic Obstructive Lung Disease
NMI: Normalized Mutual Information
FDR: False Discovery Rate
BMI: Body Mass Index
LAA: Low Attenuation Areas
HU: Hounsfield Units
Pi10: Average Wall Thickness for a Hypothetical airway of 10-mm lumen perimeter on CT
