# Supplementary material for: Identifying chronic obstructive pulmonary disease from integrative omics and clustering in lung tissue
Source: BMC Pulm Med. 2023 Apr 11;23:115. doi: 10.1186/s12890-023-02389-5 (PMC10091624; doi:10.1186/s12890-023-02389-5)
Supplement: Supplementary file 1 — Supplementary Material 1 [file 12890_2023_2389_MOESM1_ESM.docx]

Supplementary Material

**Supplemental Methods:**

**Gene Expression and DNA Methylation Data QC and Processing:**

Quality control for the gene expression data was performed to identify and remove outlier samples as well as samples with low-quality levels. The gene expression profiling data were then background-corrected, log2 transformed, and quantile normalized resulting in 32,831 probes as previously described and as represented in GEO accession GSE76925.[1] To prepare for clustering analysis, gene expression data were filtered to a minimum mean log2 expression of six to prepare for clustering analyses, resulting in 14,842 probes. Moreover, the gene expression data were then normalized to a standard normal distribution (each probe with mean expression 0 and standard deviation 1). Gene expression probes were annotated to gene symbols using the illuminaHumanv4.db (v 1.26.0) R Bioconductor package. Prior to quality control, there were 485,512 CpG sites evaluated for each of the 160 lung tissue samples. Quality control for the methylation data included detection P value filtering, removal of cross-reactive probes, and removal of probes within 0 to 1 base pairs of a known SNP (from dbSNP build 141),[2] resulting in 349,826 CpG sites and 160 lung tissue samples in the clean methylation data. Methylation probes were then background-adjusted, beta-mixture quantile (BMIQ) normalized, and then converted to percent methylation per probe, as previously described.[3] Methylation probes were annotated to the nearest transcript using the FDb.InfiniumMethylation.hg19 (v 2.2.0) R Bioconductor package. To further prepare for clustering analysis, the methylation data were filtered to include only the set of methylation probes with nearest transcript overlapping with the gene symbol from the clean gene expression probes, as above. The methylation beta values were then normalized to a standard normal distribution (each probe with mean expression 0 and standard deviation 1). These quality control and data processing steps left a total of 188,263 methylation probes for analysis. One of the gene expression batches and one of the methylation batches were comprised mostly of COPD cases, which would likely have led to confounding of batched variability with molecular differences due to COPD in our unsupervised clustering results. Thus, to reduce the potential confounding effects of technical artifacts from different batches, from the clean gene expression and methylation profiling data, we selected overlapping lung tissue samples from a single batch of gene expression and a single batch of DNA methylation data.

**Details of the Similarity Network Fusion (SNF) Method**:

We employed Similarity Network Fusion (SNF) [4] and spectral clustering [5] through the SNFtool (v 2.3.0) package in R to find lung tissue subtypes from integrated gene expression and methylation profiling. We first generated a symmetric matrix of sample-to-sample Euclidean distance independently for the cleaned and normalized gene expression and methylation data using the SNFtool dist2() function. We then tuned hyperparameters for SNF by iteratively generating similarity matrices across the range of recommended hyperparameters and chose the “optimal” set of hyperparameters (including number of neighbors [K] and scaling parameter for sample similarity [α]) resulting in the expression and methylation sample-to-sample similarity matrices with the highest variance. Then the distance matrices for the expression and methylation data were converted to sample-to-sample similarity matrices in SNFtool with the optimal number of neighbors (K) and scaling parameter (α) from the hyperparameter tuning step. The expression and methylation similarity matrices were then combined utilizing the SNFtool SNF() function with 15 iterations, which is the default. In brief, the SNF algorithm utilizes message-passing to iteratively propagate and accentuate sample-to-sample similarities that are shared across both the gene expression and methylation data and down-weight or eliminate weak sample-to-sample similarities across both data types or sample similarities observed with only a single data type. The SNF operation gives a final fused sample-to-sample similarity matrix representing the combined and shared information from gene expression and methylation data. A clustering algorithm can be applied to the final fused similarity matrix. Spectral clustering was then performed to identify k=2 clusters.[5] We used normalized mutual information (NMI) [6, 7] to assess the similarity of clustering solutions from gene expression alone, methylation alone, and combined expression and methylation data. NMI equal to 1 denotes identical clustering solutions.

**Details of the Entropy Based Consensus Clustering (ECC)** **Method**:

For comparison to SNF, we employed an alternate multiple-omic integration and clustering method, Entropy-based Consensus Clustering (ECC) which was implemented in Matlab using the code published with the manuscript.[8] Utilizing the same set of cleaned and normalized lung tissue gene expression and methylation data as SNF, we used ECC to first generate n k-means clustering solutions, varying k (the number of clusters) from 2 to 8. Here, 8 is determined by the square root of the number of available samples - in our case 78. The n varying k-means solutions are labeled “basic partitions” and the n basic partitions are generated for each set of input data and then are concatenated. In our case, the n basic partitions were generated separately for lung tissue gene expression and methylation and then were combined by the ECC method by way of an entropy-based utility function that attempts to find consensus across the 2n basic partitions. As with SNF, we set the expected number of clusters from ECC to be k=2, given the dichotomy of the lung tissue samples originating from former-smoking controls (normal spirometry) and severe COPD cases. In our initial ECC analysis, we set n=100 basic partitions for each omics data set (gene expression and methylation). We ran ECC five times and noted that five unique clustering solutions of the merged expression and methylation data were generated (Supplemental Figure S1). To increase the stability of the clustering result, we stepwise increased the n basic partitions (100 -> 2500 -> 7500 -> 15000) for the expression and methylation data, and we observed complete agreement between five final clustering solutions with n=15000 basic partitions. Thus, our final ECC clustering solution was generated by consensus from 30000 basic partitions (15000 each for the gene expression and methylation data). As with SNF, we used the NMI to assess the similarity of clustering solutions from gene expression alone, methylation alone, and combined expression and methylation data.

**Supplemental Table S1**. Demographic, clinical, and imaging features of two clusters identified from spectral clustering of similarity matrix of gene expression data from 78 lung tissue samples.

| **Feature** | **SNF Cluster 1** | **SNF Cluster 2** | **P value** |
| --- | --- | --- | --- |
| N (%) | 48 (62%) | 30 (38%) |  |
| Age | 63.01 (8.12) | 66.79 (8.52) | 0.053 |
| Male Sex, N (%) | 19 (39.6%) | 14 (46.7%) | 0.70 |
| Caucasian Race, N (%) | 43 (89.6%) | 30 (100%) | 0.18 |
| BMI | 27.66 (5.95) | 26.04 (4.65) | 0.21 |
| FEV_1_ % Predicted | 48 [22.8, 91.3] | 35.50 [21.5, 95.8] | 0.69 |
| FEV_1_/FVC Ratio | 0.57 [0.28, 0.78] | 0.39 [0.25, 0.76] | 0.15 |
| COPD, N (%) | 25 (52.1%) | 20 (66.7%) | 0.30 |
| Pack-Years Smoking | 42.24 (23.94) | 56.27 (28.18) | **0.021** |
| Months Since Quit Smoking | 66.00 [11.00, 167.50] | 108.00 [72.00, 144.00] | 0.22 |
| %LAA < -950 HU | 0.12 [0.02, 0.31] | 0.32 [0.07, 0.39] | **0.043** |
| HU at 15th % Lung Density Histogram | -937.52 (45.35) | -962.54 (30.88) | **0.027** |
| Pi10 | 4.23 (0.46) | 4.03 (0.33) | 0.19 |

Unless otherwise indicated values are either mean (standard deviation) or median [interquartile range].

*P value is from t-test for variables expressed as mean (sd) and Wilcoxon rank sum test for variables expressed as median [IQR]

BMI = body mass index; LAA = lung attenuation area; HU = Hounsfield units; Pi10 = square root of cross-sectional area of hypothetical 10mm internal perimeter airway

**Supplemental Table S2**. Demographic, clinical, and imaging features of two clusters identified from spectral clustering of similarity matrix of DNA methylation data from 78 lung tissue samples.

| **Feature** | **SNF Cluster 1** | **SNF Cluster 2** | **P value** |
| --- | --- | --- | --- |
| N (%) | 30 (38%) | 48 (62%) |  |
| Age | 63.92 (8.87) | 64.81 (8.21) | 0.65 |
| Male Sex, N (%) | 14 (46.7%) | 19 (39.6%) | 0.70 |
| Caucasian Race, N (%) | 27 (90.0%) | 46 (95.8%) | 0.58 |
| BMI | 27.43 (6.11) | 26.78 (5.13) | 0.61 |
| FEV_1_ % Predicted | 31.50 [22.25, 82.00] | 65.50 [23.50, 99.50] | **0.030** |
| FEV_1_/FVC Ratio | 0.34 [0.25, 0.77] | 0.65 [0.29, 0.77] | 0.18 |
| COPD, N (%) | 21 (70%) | 24 (50%) | 0.13 |
| Pack-Years Smoking | 47.97 (22.09) | 47.44 (28.96) | 0.93 |
| Months Since Quit Smoking | 72 [24, 108] | 116 [31.5, 228] | 0.15 |
| %LAA < -950 HU | 0.24 [0.07, 0.33] | 0.17 [0.02, 0.39] | 1 |
| HU at 15th % Lung Density Histogram | -949.18 (40.42) | -947.49 (42.72) | 0.88 |
| Pi10 | 4.11 (0.46) | 4.16 (0.39) | 0.77 |

Unless otherwise indicated values are either mean (standard deviation) or median [interquartile range].

*P value is from t-test for variables expressed as mean (sd) and Wilcoxon rank sum test for variables expressed as median [IQR]

BMI = body mass index; LAA = lung attenuation area; HU = Hounsfield units; Pi10 = square root of cross-sectional area of hypothetical 10mm internal perimeter airway

**Supplemental Table S3**. Demographic, clinical, and imaging features of two clusters identified from Entropy-based Consensus Clustering (ECC) of gene expression data from 78 lung tissue samples.

| **Feature** | **ECC Cluster 1** | **ECC Cluster 2** | **P value** |
| --- | --- | --- | --- |
| N (%) | 38 (49%) | 40 (51%) |  |
| Age | 66.5 (7.9) | 62.6 (8.5) | **0.040** |
| Male Sex, N (%) | 18 (47.4%) | 15 (37.5%) | 0.51 |
| Caucasian Race, N (%) | 38 (100%) | 35 (87.5%) | 0.073 |
| BMI | 26.1 (4.6) | 27.9 (6.2) | 0.17 |
| FEV_1_ % Predicted | 35.5 [23.5, 95.75] | 65.5 [21.75, 91.25] | 0.94 |
| FEV_1_/FVC Ratio | 0.39 [0.27, 0.77] | 0.63 [0.29, 0.78] | 0.27 |
| COPD, N (%) | 25 (65.8%) | 20 (50%) | 0.24 |
| Pack-Years Smoking | 58.1 (30.8) | 37.7 (16.2) | **0.00043** |
| Months Since Quit Smoking | 108 [60, 144] | 60 [9, 160.5] | 0.17 |
| %LAA < -950 HU | 0.30 [0.06, 0.39] | 0.12 [0.02, 0.31] | 0.15 |
| HU at 15th % Lung Density Histogram | -958.7 (32.4) | -936.9 (47.4) | 0.052 |
| Pi10 | 4.08 (0.39) | 4.19 (0.43) | 0.48 |

Unless otherwise indicated values are either mean (standard deviation) or median [interquartile range].

*P value is from t-test for variables expressed as mean (sd) and Wilcoxon rank sum test for variables expressed as median [IQR]

BMI = body mass index; LAA = lung attenuation area; HU = Hounsfield units; Pi10 = square root of cross-sectional area of hypothetical 10mm internal perimeter airway

**Supplemental Table S4**. Demographic, clinical, and imaging features of two clusters identified from Entropy-based Consensus Clustering (ECC) of DNA methylation data from 78 lung tissue samples.

| **Feature** | **ECC Cluster 1** | **ECC Cluster 2** | **P value** |
| --- | --- | --- | --- |
| N (%) | 22 (28%) | 56 (72%) |  |
| Age | 63.3 (8.1) | 64.9 (8.6) | 0.43 |
| Male Sex, N (%) | 11 (50%) | 22 (39.3%) | 0.54 |
| Caucasian Race, N (%) | 19 (86.4%) | 54 (96.4%) | 0.26 |
| BMI | 27.8 (6.8) | 26.7 (4.9) | 0.43 |
| FEV_1_ % Predicted | 30.5 [20.5, 38] | 82 [23.75, 99.5] | **0.0059** |
| FEV_1_/FVC Ratio | 0.32 [0.24, 0.39] | 0.71 [0.29, 0.78] | **0.013** |
| COPD, N (%) | 18 (81.8%) | 27 (48.2%) | **0.014** |
| Pack-Years Smoking | 51.5 (23) | 46.1 (27.7) | 0.42 |
| Months Since Quit Smoking | 54 [14.25, 107] | 112 [36, 201] | 0.057 |
| %LAA < -950 HU | 0.28 [0.11, 0.34] | 0.17 [0.02, 0.38] | 0.50 |
| HU at 15th % Lung Density Histogram | -953.2 (40.6) | -946.1 (42.1) | 0.57 |
| Pi10 | 4.20 (0.44) | 4.10 (0.40) | 0.55 |

Unless otherwise indicated values are either mean (standard deviation) or median [interquartile range].

*P value is from t-test for variables expressed as mean (sd) and Wilcoxon rank sum test for variables expressed as median [IQR]

BMI = body mass index; LAA = lung attenuation area; HU = Hounsfield units; Pi10 = square root of cross-sectional area of hypothetical 10mm internal perimeter airway

**Supplemental Table S5**. Reactome pathways significantly enriched in SNF cluster differentially expressed transcripts.

| ID | Description | p.adjust |
| --- | --- | --- |
| R-HSA-6798695 | Neutrophil degranulation | 8.6E-17 |
| R-HSA-983705 | Signaling by the B Cell Receptor (BCR) | 2.2E-04 |
| R-HSA-168898 | Toll-like Receptor Cascades | 2.2E-04 |
| R-HSA-449147 | Signaling by Interleukins | 1.4E-03 |
| R-HSA-198933 | Immunoregulatory interactions between a Lymphoid and a non-Lymphoid cell | 5.2E-03 |
| R-HSA-1168372 | Downstream signaling events of B Cell Receptor (BCR) | 6.2E-03 |
| R-HSA-2029480 | Fcgamma receptor (FCGR) dependent phagocytosis | 1.2E-02 |
| R-HSA-5668599 | RHO GTPases Activate NADPH Oxidases | 1.3E-02 |
| R-HSA-3299685 | Detoxification of Reactive Oxygen Species | 1.4E-02 |
| R-HSA-166016 | Toll Like Receptor 4 (TLR4) Cascade | 1.7E-02 |
| R-HSA-202424 | Downstream TCR signaling | 2.0E-02 |
| R-HSA-202403 | TCR signaling | 2.2E-02 |
| R-HSA-622312 | Inflammasomes | 2.4E-02 |
| R-HSA-6783783 | Interleukin-10 signaling | 2.6E-02 |
| R-HSA-2454202 | Fc epsilon receptor (FCERI) signaling | 2.6E-02 |
| R-HSA-216083 | Integrin cell surface interactions | 2.6E-02 |
| R-HSA-168643 | Nucleotide-binding domain, leucine rich repeat containing receptor (NLR) signaling pathways | 2.8E-02 |
| R-HSA-5663205 | Infectious disease | 2.8E-02 |
| R-HSA-5621575 | CD209 (DC-SIGN) signaling | 2.9E-02 |
| R-HSA-168188 | Toll Like Receptor TLR6:TLR2 Cascade | 3.1E-02 |
| R-HSA-202733 | Cell surface interactions at the vascular wall | 3.1E-02 |
| R-HSA-975138 | TRAF6 mediated induction of NFkB and MAP kinases upon TLR7/8 or 9 activation | 3.1E-02 |
| ID | Description | p.adjust |
| R-HSA-9006335 | Signaling by Erythropoietin | 3.1E-02 |
| R-HSA-1679131 | Trafficking and processing of endosomal TLR | 3.1E-02 |
| R-HSA-202427 | Phosphorylation of CD3 and TCR zeta chains | 3.1E-02 |
| R-HSA-2029482 | Regulation of actin dynamics for phagocytic cup formation | 3.1E-02 |
| R-HSA-168138 | Toll Like Receptor 9 (TLR9) Cascade | 3.1E-02 |
| R-HSA-162587 | HIV Life Cycle | 3.1E-02 |
| R-HSA-202430 | Translocation of ZAP-70 to Immunological synapse | 3.1E-02 |
| R-HSA-168181 | Toll Like Receptor 7/8 (TLR7/8) Cascade | 3.1E-02 |
| R-HSA-162906 | HIV Infection | 3.1E-02 |
| R-HSA-2132295 | MHC class II antigen presentation | 3.1E-02 |
| R-HSA-181438 | Toll Like Receptor 2 (TLR2) Cascade | 3.1E-02 |
| R-HSA-1222556 | ROS and RNS production in phagocytes | 3.1E-02 |
| R-HSA-5663213 | RHO GTPases Activate WASPs and WAVEs | 3.1E-02 |
| R-HSA-381119 | Unfolded Protein Response (UPR) | 3.1E-02 |
| R-HSA-446203 | Asparagine N-linked glycosylation | 3.3E-02 |
| R-HSA-5675221 | Negative regulation of MAPK pathway | 3.3E-02 |
| R-HSA-937061 | TRIF(TICAM1)-mediated TLR4 signaling | 3.3E-02 |
| R-HSA-1169091 | Activation of NF-kappaB in B cells | 3.5E-02 |
| R-HSA-2424491 | DAP12 signaling | 3.7E-02 |
| R-HSA-499943 | Interconversion of nucleotide di- and triphosphates | 3.7E-02 |
| R-HSA-432722 | Golgi Associated Vesicle Biogenesis | 3.9E-02 |
| R-HSA-199992 | trans-Golgi Network Vesicle Budding | 4.1E-02 |
| R-HSA-75955 | RNA Polymerase II Transcription Elongation | 4.8E-02 |

**Supplemental Table S6**. Reactome pathways significantly enriched in ECC cluster differentially expressed transcripts.

| ID | Description | p.adjust |
| --- | --- | --- |
| R-HSA-198933 | Immunoregulatory interactions between a Lymphoid and a non-Lymphoid cell | 7.1E-06 |
| R-HSA-202733 | Cell surface interactions at the vascular wall | 1.2E-02 |
| R-HSA-6798695 | Neutrophil degranulation | 1.9E-02 |
| R-HSA-449147 | Signaling by Interleukins | 3.0E-02 |
| R-HSA-202433 | Generation of second messenger molecules | 4.6E-02 |

**Supplemental Figure S1**. Normalized mutual information (NMI) between 5 subsequent clustering solutions for the lung tissue data using ECC with 100 (default) basic partitions for each single omics data type (gene expression and methylation). 5 unique clustering solutions were generated.

**Supplemental References**

1. Morrow JD, Zhou X, Lao T, Jiang Z, DeMeo DL, Cho MH, Qiu W, Cloonan S, Pinto-Plata V, Celli B, Marchetti N, Criner GJ, Bueno R, Washko GR, Glass K, Quackenbush J, Choi AM, Silverman EK, Hersh CP. Functional interactors of three genome-wide association study genes are differentially expressed in severe chronic obstructive pulmonary disease lung tissue. *Scientific reports* 2017: 7: 44232.

2. Database of Single Nucleotide Polymorphisms (dbSNP). Bethesda, MD: National Center for Biotechnology Information, National Library of Medicine. (dbSNP Build ID: 141). URL: http://www.ncbi.nlm.nih.gov/SNP/. [cited; Available from: http://www.ncbi.nlm.nih.gov/SNP/

3. Morrow JD, Cho MH, Hersh CP, Pinto-Plata V, Celli B, Marchetti N, Criner G, Bueno R, Washko G, Glass K, Choi AM, Quackenbush J, Silverman EK, DeMeo DL. DNA methylation profiling in human lung tissue identifies genes associated with COPD. *Epigenetics* 2016: 1-10.

4. Wang B, Mezlini AM, Demir F, Fiume M, Tu Z, Brudno M, Haibe-Kains B, Goldenberg A. Similarity network fusion for aggregating data types on a genomic scale. *Nature methods* 2014: 11(3): 333-337.

5. Ng AY, Jordan MI, Weiss Y. On spectral clustering: analysis and an algorithm. Proceedings of the 14th International Conference on Neural Information Processing Systems: Natural and Synthetic. MIT Press, Vancouver, British Columbia, Canada, 2001; pp. 849-856.

6. Kuncheva LI, Hadjitodorov ST. Using diversity in cluster ensembles. In: 2004 IEEE International Conference on Systems, Man and Cybernetics (IEEE Cat No04CH37583); 2004 10-13 Oct. 2004; 2004. p. 1214-1219 vol.1212.

7. Danon L, Díaz-Guilera A, Duch J, Arenas A. Comparing community structure identification. *Journal of Statistical Mechanics: Theory and Experiment* 2005: 2005(09): P09008-P09008.

8. Liu H, Zhao R, Fang H, Cheng F, Fu Y, Liu YY. Entropy-based consensus clustering for patient stratification. *Bioinformatics* 2017: 33(17): 2691-2698.
